# Depression and Anxiety Mediate the Relationship between Discrimination and Well-Being in a Sample of Latinx Adults with Type 2 Diabetes: Results from a Dual Mediation Analysis

**DOI:** 10.1007/s10903-024-01582-w

**Published:** 2024-01-20

**Authors:** Kevin A. Matlock, Rafael Pérez-Escamilla, Julie Wagner

**Affiliations:** 1 Department of Psychiatry, University of Oxford, Oxford, UK; 2 Worcester College, University of Oxford, Oxford, UK; 3 Yale School of Public Health, Yale University, New Haven, CT USA; 4 University of Connecticut Health Center, University of Connecticut, Farmington, CT USA

**Keywords:** Anxiety, Depression, Discrimination, Latinx, Type 2 diabetes, Well-being

## Abstract

Latinxs experience greater risk for type 2 diabetes, discrimination, and poor mental health. The pathways linking these factors, however, are not well understood. This study tested whether depression and anxiety mediated the relationship between discrimination and well-being. Bootstrapped mediation tests were conducted using a sample of Latinx adults with type 2 diabetes (*n* = 121) and regression models adjusted for demographic and health covariates. Depression and anxiety fully and jointly mediated the effect of discrimination on well-being; everyday discrimination was linked to elevated symptoms of depression and anxiety which were, in turn, independently linked to reduced emotional well-being. Moreover, the effect size for the anxiety pathway (β=–0.13) was 60% larger than for depression (β=–0.08). Dual mediation suggests depression, and especially anxiety, may be important targets for interventions seeking to mitigate the deleterious effects of discrimination. Findings have important implications for psychotherapeutic treatments and public health policy.

## Background

*Latinx*, a gender-inclusive term for persons who identify as Hispanic or Latina/Latino, refers to more than 60 million people, making it the largest minoritized ethnic group in the US [1, 2]. The prevalence of type 2 diabetes in this population (13%) is nearly double that of non-Hispanic White persons [3]. Latinxs also face a greater risk of poor mental health. The odds of developing panic disorder or generalized anxiety disorder are 70% higher for Latinxs than non-Hispanic White persons [4], while the odds of developing depressive symptoms are 90% higher [5]. Given these challenges, fostering a better understanding of how Latinxs’ experience diabetes and mental health is crucial.

## Conceptual Framework

Latinxs and persons with type 2 diabetes both report lower subjective well-being [6–8]. This disparity may be at least partially attributable to discrimination. Ethnic and racial discrimination, in particular *everyday discrimination*—relatively small, day-to-day occurrences like disrespectful speech, insults, and poorer service at restaurants [9]—is reported by a third of Latinxs [10]. Though seemingly innocuous, these events can have a profound impact on mental health comparable to more overt forms of discrimination like threats of violence [11]. Indeed, Latinxs who encounter frequent everyday discrimination report greater stress and more severe depression [12]—factors associated with diminished well-being [13, 14]. Likewise, illness-based discrimination can lead to similar reductions in well-being for persons with diabetes [15].

Evidence suggests discrimination may undermine well-being by intensifying negative mood states [12, 16, 17]. However, the pathways underlying this relationship are not well understood and have yet to be explored in Latinx persons with type 2 diabetes. Therefore, this study aimed to perform a secondary analysis using cross-sectional data from a sample of Latinx adults with type 2 diabetes to test whether symptoms of depression and anxiety mediated the relationship between everyday discrimination and emotional well-being.

## Method

### Participants

Baseline data were sourced from the Community health educators Assisting Latinos Manage Stress and Diabetes (CALMS-D) study [18] (ClinicalTrials.gov identifier NCT01578096). Participants in CALMS-D were recruited for a stress-management intervention from an urban outpatient clinic in Hartford, Connecticut, US. Participants were included if they self-identified as Latino/Latina or Hispanic and were ambulatory; chart review confirmed participants were adults with type 2 diabetes (duration ≥ 6 months). Participants were excluded from CALMS-D based on medical records or self-reports indicating (a) medical instability or intensive treatment, (b) bipolar disorder or thought disorder, (c) attempted suicide or psychiatric hospitalization in the past 2 years, or (d) substance use disorder.

### Data Collection

Eligible participants were interviewed in their homes by trained and supervised community health workers in Spanish or English based on the participant’s preference. Self-report responses were recorded using Remote Electronic Data Capture (REDCap) [19], an internet survey tool. Blood samples were taken during home visits and transported to a university laboratory for analysis. Participants were paid 10 USD for each interview and for each laboratory assessment. For the full CALMS-D protocol, see Wagner and colleagues [18].

#### Ethical Approval

CALMS-D was conducted in accordance with the Helsinki Declaration and was approved by Institutional Review Boards of participating institutions. Participants signed written informed consent forms in their preferred language. The study was co-designed and implemented through an equitable partnership between UCONN Health, Yale University, Hartford Hospital, and the Hispanic Health Council.

### Measures

#### Covariates

Gender, age, monthly household income (USD), and perceived health on a scale from 1 (*poor*) to 5 (*excellent*) were assessed using single-item questions. Smoking status and time spent exercising (number of days last week engaged in at least 30 min of physical activity) were assessed using relevant single items from the Summary of Diabetes Self-Care Activities (SDSCA) scale [20]. Laboratory assessments of fasting serum glucose and insulin levels were used to calculate insulin resistance using the HOMA-IR formula, where higher values indicate greater impairment of insulin function [21, 22].

#### Predictor

Everyday discrimination was measured using the 10-item Everyday Discrimination Scale (EDS) [9]. The EDS includes items such as “people act as if they are afraid of you” and “you are followed around in stores”. For each item, participants reported the number of times they had experienced discrimination in day-to-day life on a scale from 0 (*Never*) to 4 (*Four or more times*). The EDS has been validated in English [9] and Spanish [23].

#### Mediators

Depression was measured using the 8-item Patient Health Questionnaire (PHQ-8) [24]. The PHQ-8 assesses how often over the last two weeks participants experienced symptoms of depression using items like “feeling down, depressed, or hopeless” and “feeling tired or having little energy”. Responses were rated on a scale from 0 (*Not at all*) to 3 (*Nearly every day*). Anxiety was measured using the 8-item Patient-Reported Outcomes Measurement Information System (PROMIS) anxiety short-form scale [25]. PROMIS assesses how often over the last seven days participants experienced symptoms of anxiety using items like “felt nervous”, “felt tense”, and “felt fearful”. Responses were rated on a scale from 0 (*No, never*) to 4 (*Always*). The PHQ-8 and PROMIS have been validated in English [24, 25] and Spanish [26, 27].

#### Outcome

Emotional well-being was measured using the 5-item World Health Organization (WHO-5) well-being index [28]. The WHO-5 assesses well-being over the last two weeks using items like “enjoyed your daily activities”, woke up “feeling fresh and rested”, and “felt your life was filled with things that interest you”. Responses were rated on a scale from 0 (*No, at no time*) to 4 (*All of the time*). The WHO-5 has been validated in English [28] and Spanish [23, 29].

### Analysis

Categorical covariates were dichotomized for analysis. Reference groups for dichotomous variables were female gender (vs. male), monthly household income at or below 1,000 USD (vs. above), perceived health rated as fair or poor (vs. good, great, or excellent), and being a non-smoker (vs. current smoker). Continuous variables retained their original coding, with lower values reflecting younger age, less frequent exercise, lower insulin resistance, less frequent discrimination, less severe depression, less severe anxiety, and lower emotional well-being.

Analyses were conducted in STATA 16 [30]. Cross-sectional relationships were evaluated using baseline values for all measures. Bivariate relationships were analyzed using Pearson’s correlation. Direct effects were analyzed using multiple regression with discrimination, depression, and anxiety entered as predictors; well-being entered as the outcome; and gender, income, perceived health, smoking status, age, exercise, and insulin resistance entered as covariates. All covariates were selected based on sample size constraints and potential to act as confounds, as previous studies have reported associations between these factors and both discrimination [10, 11, 31, 32] and well-being [33–38]. Glycemic control (i.e., HbA1c) was initially considered as a covariate in place of insulin resistance, but the latter was chosen for the final model because it shared a significant correlation with at least one other variable in the model, and is considered a more reliable indicator of long-term metabolic health [39].

For the final regression model, all continuous variables were centered and standardized. Normality of residuals and heteroscedasticity were tested using visual inspection of Q-Q plots and a fitted-value plot, respectively. Multicollinearity was examined using variance inflation factor (VIF) values. Indirect pathways and mediation effects were tested using a dual-mediation model described by Preacher and Hayes [40], a technique which generalizes the Baron and Kenny approach to multiple mediators. In addition, significance testing relied on bootstrapping to generate robust bias-corrected confidence intervals for indirect effects to adjust for potentially non-normal residuals [41].

## Results

### Sample

The sample consisted of 121 Latinx adults aged 21–86 years old (*M* = 61) with type 2 diabetes. Participants were predominately women (74%) with less than a high-school education (77%). All were first-generation migrants who had lived in the US for less than 6 years (*M* = 2.9). Most (67%) had a monthly income near or below the federal poverty threshold for a single-person household (approximately 1000 USD) [42]. Insulin resistance was high, with a large majority (77%) exceeding the median for the uppermost quintile in the general population (HOMA-IR > 4.1) [43]. Half the sample (46%) did not meet recommendations for physical activity in the previous week (30 min per day) [44].

A majority of the sample (54%) reported experiencing everyday discrimination. Among these individuals, discrimination was most frequently attributed to race (34%) and difficulty speaking English (11%), though several indicated an unspecified (22%) or unknown (13%) source. Several participants (29%) had depression totals above the clinical threshold (PHQ ≥ 10) [24], while a majority (65%) had anxiety scores that matched or exceeded estimates for clinical populations on at least one PROMIS item, and several (37%) had clinically significant anxiety on at least half the items [45]. A third of the sample had well-being totals below the threshold deemed clinically significant (WHO-5 < 52%) [46]. Additional sample characteristics are listed in Table 1.

### Correlation Analysis

Correlations for untransformed variables (Table 1) revealed that well-being was inversely associated with more frequent discrimination, *r*(118)=–0.33, *p* < .001, as well as more severe symptoms of both depression, *r*(117)=–0.65, *p* < .001, and anxiety, *r*(117)=–0.71, *p* < .001. Only two covariates were not associated with well-being: gender, *r*(117) = 0.02, *p* = .82, and insulin resistance, *r*(113) =–0.01, *p* = .92.

**Table 1:** Sample characteristics and pairwise correlations

Variable	Mode (%) or M (SD)	1	2	3	4	5	6	7	8	9	10
1. Gender^a^	Women (74%)										
2. Income^a^	$1000 or less (67%)	0.11									
3. Health^a^	Poor or fair (60%)	0.01	0.13								
4. Smoker^a^	No (82%)	0.16	0.12	–0.04							
5. Age (years)	60.8 (11.7)	–0.06	–0.17	0.07	–0.27**						
6. Exercise (days)	4.4 (3.0)	–0.02	–0.01	0.08	–0.07	0.08					
7. HOMA-IR	13.0 (13.8)	0.21*	0.25**	0.07	0.00	–0.13	–0.02				
8. EDS	0.5 (0.7)	0.02	0.17	–0.26**	0.17	–0.39***	0.07	0.19*			
9. PHQ-8	6.2 (5.5)	–0.06	–0.02	–0.40***	0.30***	–0.17	–0.06	0.18	0.37***		
10. PROMIS	15.0 (7.6)	–0.10	–0.14	–0.36***	0.21*	–0.17	–0.17	0.10	0.32***	0.76***	
11. WHO-5	62.3 (29.6)	0.02	0.19*	–0.37***	–0.26**	0.33***	0.25**	–0.01	–0.33***	–0.65***	–0.71***

*Note*: Descriptives are given as mode (% of sample) for dichotomous variables and mean (standard deviation) for continuous variables

*Abbreviations*: EDS, Everyday Discrimination Scale; HOMA-IR; Homeostatic Model Assessment of Insulin Resistance; PHQ-8, Patient Health Questionnaire 8-item; PROMIS, Patient-Reported Outcomes Measurement Information System scale; WHO-5, World Health Organization 5-item well-being index

* *p* < .05, ** *p* < .01, *** *p* < .001

^a^Dichotomous variable; reference groups are women (vs. men), monthly income of $1000 or less (vs. higher than $1000), poor or fair health (vs. good or better), and non-smoker (vs. smoker)

### Regression Analysis

#### Assumptions and Transformations

Visual inspection of Q-Q plots revealed minor normality violations for residuals. While Monte Carlo simulations suggest regression models remain robust even for serious violations [47], non-normal variables in the regression model were adjusted using transformations to improve normality. A positive power transformation was employed for well-being to correct for positive skewness, and inverse power transformations were employed to correct for negative skewness in discrimination, anxiety, and depression. To aid interpretation and preserve directionality of the original measures, signs were flipped (e.g., positive to negative) for variables using an inverse transformation.

Responses did not exceed three standard deviations from center for any variable, indicating no outliers were present. Following transformations, visual inspection of scatterplots between each predictor and the outcome revealed no non-linear relationships. A fitted-value plot showed a roughly uniform pattern, suggesting no serious problems with heteroscedasticity. This was confirmed via a non-significant Cameron and Trivedi decomposition test, χ^2^(50) = 43.7, *p* = .72. Variance inflation factor (VIF) values ranged from a minimum of 1.0 (exercise) to a maximum of 2.4 (depression); VIF for all predictors and covariates fell below 10, signifying no problems with multicollinearity.

#### Direct Effects

As a set, predictors and covariates in the final regression model explained more than a third of the total variation in well-being, *R*^2^ = 0.36, *F*(8,105) = 7.4, *p* < .001. Transformations made unstandardized slopes and confidence intervals difficult to interpret, so unique associations between each variable and the outcome were described using standardized slopes and *p*-value thresholds (Table 2). Slopes for both predictors, depression and anxiety, were independently associated with well-being, *ps* < 0.01, with the effect of anxiety rating about 50% stronger than the effect of depression. After controlling for other factors, the slope or the remaining predictor, everyday discrimination, was not uniquely associated with well-being, *p* = .28.

**Table 2:** Regression model predicting well-being (WHO-5)

Variables (*n* = 115)	β	t	p
*Covariates*			
Gender^a^	–0.03	0.4	0.68
Income^a^	0.18	2.7	0.01*
Health^a^	0.06	0.8	0.41
Smoker^a^	–0.05	0.8	0.45
Age (years)	0.20	3.0	< 0.01**
Exercise (days)	0.11	1.8	0.07
Insulin resistance (HOMA-IR)	0.10	1.5	0.13
*Predictors*			
Discrimination (EDS)	–0.08	1.1	0.28
Depression (PHQ-8)	–0.25	2.7	0.01*
Anxiety (PROMIS)	–0.38	4.2	< 0.01**

*Abbreviations*: EDS, Everyday Discrimination Scale; HOMA-IR; Homeostatic Model Assessment of Insulin Resistance; PHQ-8, Patient Health Questionnaire 8-item; PROMIS, Patient-Reported Outcomes Measurement Information System scale; WHO-5, World Health Organization 5-item well-being index

* *p* < .05, ** *p* < .01

^a^Dichotomous variable; reference groups are women (vs. men), monthly income of 1000 USD or less (vs. higher than 1000 USD), poor or fair health (vs. good or better), and non-smoker (vs. smoker)

#### Mediation and Indirect Effects

The predictor (discrimination) shared a significant correlation with both potential mediators (depression and anxiety) and all three variables were significantly correlated with the outcome (well-being). This pattern of association fulfilled the criteria necessary for mediation [40]. Moreover, the absence of a significant effect for discrimination in the final regression model suggested one or both mediators masked the direct effect, further supporting the likelihood of mediation [48].

**Fig. 1:**
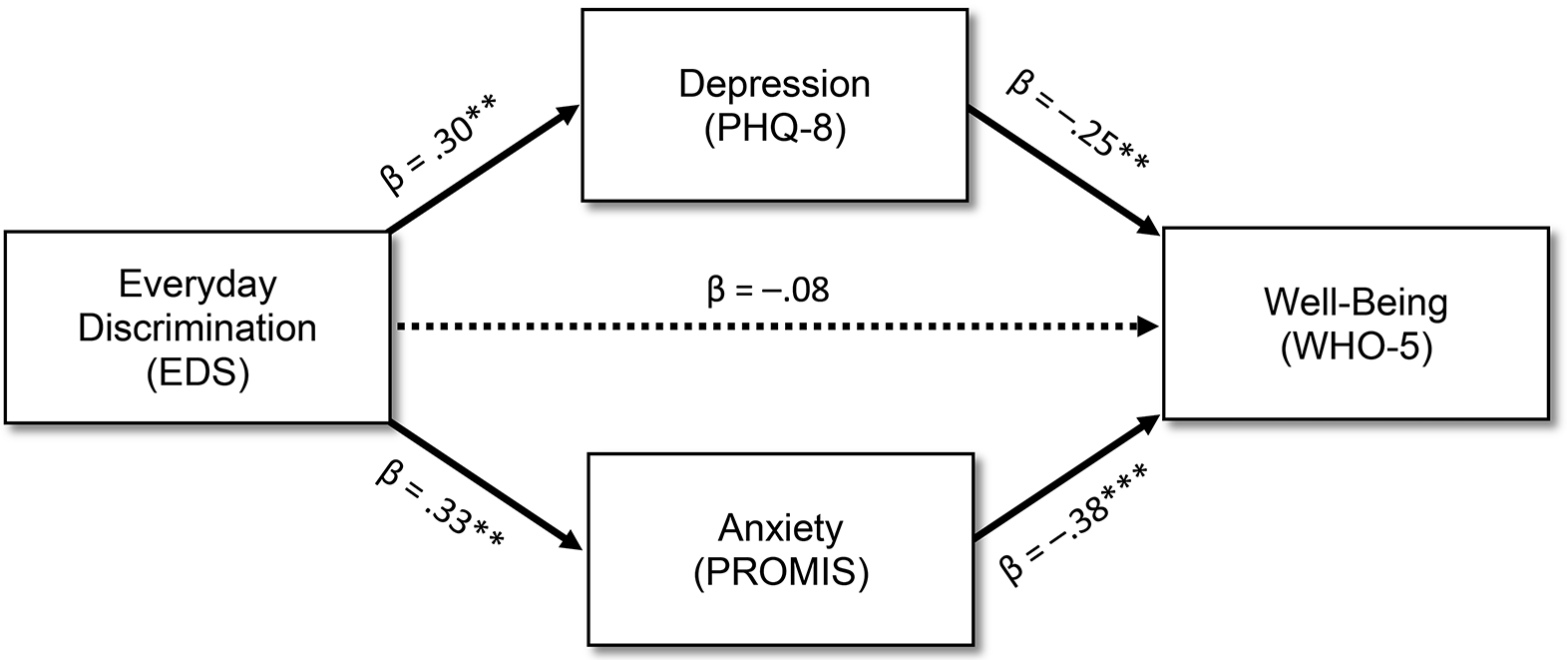
Dual-mediation model. *Abbreviations*: EDS, Everyday Discrimination Scale; PHQ-8, Patient Health Questionnaire 8-item; PROMIS, Patient-Reported Outcomes Measurement Information System scale; WHO-5, World Health Organization 5-item well-being index. * *p* < .05, ** *p* < .01, *** *p* < .001

To test whether the effect of discrimination on well-being was mediated by depression and anxiety, a dual-mediation analysis was performed following the process outlined by Preacher and Hayes [40]. Standardized estimates and significance tests of pathway segments were drawn from the full regression model, or from covariate-adjusted regression models of the predictor and each mediator entered as the outcome (Fig. 1). Indirect pathways were calculated by taking the product of pathway segments. Following bootstrapping with 1,000 repetitions, significance testing using robust bias-corrected confidence intervals revealed that the indirect, mediated pathways from discrimination to well-being through depression, β=–0.08; through anxiety, β=–0.13; and the sum of these two pathways, β=–0.20, were all significant, *p*s < 0.05. Meanwhile, the remaining direct, non-mediated pathway from discrimination to well-being was not significant, β=–0.08, *p* > .05.

Bias-corrected results suggest that the effect of self-reported frequency of everyday discrimination was fully and jointly mediated by symptoms of depression and anxiety. In total, more than 70% of the total effect was mediated by a combination of the pathways through depression and anxiety. Effect sizes showed that these pathways were not equally impactful, however, as the ratio of the size of the indirect pathway through depression was similar to the direct effect (0.95), while the indirect pathway through anxiety was substantially larger than the direct effect (1.57).

## Discussion

Findings from a secondary analysis of cross-sectional data for Latinx adults with type 2 diabetes supported a dual-mediation model in which the relationship between self-reported frequency of everyday discrimination and level of emotional well-being was mediated by the severity of depression and anxiety symptoms. Statistical models controlled for a variety of potential confounds, including gender, income, perceived health, smoking status, age, physical activity, and degree of insulin resistance, indicating that mediation effects were not attributable to these factors.

Together, discrimination, depression, anxiety, and the demographic and health covariates included in the full regression model explained more than one-third of the total variation in scores for well-being. The pattern of associations observed for pair-wise correlations, meanwhile, matched the criteria necessary for mediation (see Preacher & Hayes [40]); more frequent everyday discrimination (the predictor) was associated with more severe symptoms of depression and anxiety (the mediators), and all three were associated with poorer ratings for emotional well-being (the outcome). Follow-up tests using covariate-adjusted regression models and robust bias-corrected confidence intervals confirmed the presence of two significant, independent mediation pathways, such that symptoms of depression and anxiety fully and jointly mediated the effect of self-reported discrimination on well-being. These results suggest that, at least for Latinx adults with type 2 diabetes, experiences with everyday discrimination (e.g., insults, poorer service) erode a person’s sense of well-being in two distinct ways: first by increasing fear and anxiety, and second by increasing weariness and depression.

To our knowledge, this study is the first of its kind to explore this model in a Latinx sample with type 2 diabetes. However, mechanisms identified in other populations may help to explain why symptoms of depression and anxiety may act to mediate the relationship between discrimination and well-being. For instance, Velez and Moradi [49] found evidence to suggest that the relationship between heterosexist discrimination and well-being is mediated by the internalization of negative stereotypes. Given that negative schemas about the self can lead to disruptions in mood [16], it is reasonable to speculate that, in the current study, everyday discrimination may have fostered a more negative self-concept, thereby exacerbating symptoms of depression and anxiety. Another possibility is that discrimination negatively impacts affectivity by modifying a person’s beliefs about others. According to Nielsen [17], sexist and racist remarks instill feelings of both apprehension and sadness by suggesting that other people pose a threat to personal safety and lack compassion and understanding, respectively. Regardless of the underlying mechanisms, the current study and previous research indicate that discrimination increases negative affectivity which, in turn, undercuts overall emotional well-being [50, 51].

### New Contributions to the Literature

The dual-mediation model presented in this study makes several contributions for understanding the unique challenges faced by Latinxs. First, it suggests that more frequent everyday discrimination likely plays adds to the high levels of anxiety disorders [4] and depression symptomology [5] observed in this population. Second, the presence of complete, joint mediation is the first evidence of its kind to indicate discrimination undermines well-being primarily by increasing negative affectivity—a finding consistent with the well-being negativity bias reported elsewhere [51]. Third, the effect size for the anxiety pathway was 60% larger than the depression pathway, suggesting that symptoms of anxiety are likely to be especially important target areas for interventions aiming to lessen the impact of discrimination in Latinx adults with type 2 diabetes.

### Limitations and Next Steps

Findings were limited in three ways. First, this study relied on a small sample from a single clinic, limiting generalizability to other groups with different characteristics. Additionally, data were collected in 2015 and—given that discrimination is a social phenomenon influenced by societal trends—this may affect generalizability over time. For instance, the recent focus on racial and ethnic discrimination in the US may produce even more robust findings. Second, everyday discrimination was captured using a generic measure that did not explicitly account for illness-based discrimination attributable to diabetes, which may explain why a third of the sample attributed their experience with discrimination to either an unspecified or unknown source [15]. Third, despite the well-established harms caused by discrimination [17], reverse causation may explain at least a portion of the mediation effects observed in this study. For instance, previous research has found that greater negative affectivity increases the likelihood that participants will report experiencing discrimination [52], and can prime participants to interpret ambiguous scenarios as discriminatory [53]. Thus, it may be the case that diminished emotional well-being, more severe depression, or elevated anxiety-related vigilance may increase awareness or heighten perceptions of discrimination.

In light of these limitations, further research is needed to (a) confirm the experimental validity of the results from the present study; (b) determine generalizability to other populations; (c) differentiate between different sources of discrimination; (d) examine the role of internalized stereotypes and beliefs about others in mediating these effects; (e) assess the influence of social support and immigration status; (f) establish directionality using a large-sample, longitudinal study; and (g) test well-being interventions explicitly targeting symptoms of depression and anxiety.

## Conclusion

As the largest ethnic minority in the US, Latinxs face a number of challenges, including a greater risk for type 2 diabetes, more frequent discrimination, poorer mental health, and diminished emotional well-being. Findings from this study help to explain the complex relationships between these factors by supporting a dual-mediation model whereby the association between everyday discrimination and well-being is jointly mediated by symptoms of anxiety and depression. Support for this model, in particular the greater relative effect for the anxiety pathway, has important implications for future intervention studies and public health policy.
